# Supplementary material for: Theoretical Investigation of the Effects of Aldehyde Substitution with Pyran Groups in D-π-A Dye on Performance of DSSCs
Source: Molecules. 2024 Sep 3;29(17):4175. doi: 10.3390/molecules29174175 (PMC11397415; doi:10.3390/molecules29174175)
Supplement: Supplementary file 1 [file molecules-29-04175-s001.zip › molecules-3176359-supplementary.pdf]

Table S1. The maximum absorption wavelength of BC determined using the TD-DFT approach with different models.

Functionals Methods	Calculated	Experimental
B3LYP	463 nm	432 nm
B3PW91	459 nm	432 nm
CAM-B3LYP	357 nm	432 nm
MPW1PW91	435 nm	432 nm

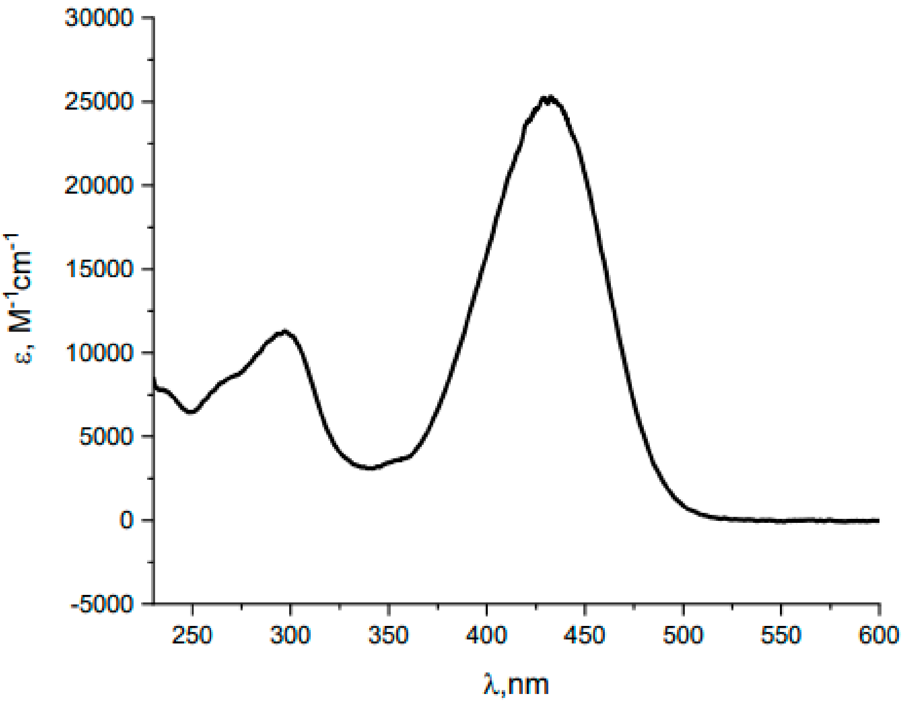

Figure S1. UV-Vis spectrum of BC in DCM solution.
